# Microstructure, Mechanical and Tribological Properties of High-Entropy Carbide (MoNbTaTiV)C_5_

**DOI:** 10.3390/ma16114115

**Published:** 2023-05-31

**Authors:** Shubo Zhang, Falian Qin, Maoyuan Gong, Zihao Wu, Meiling Liu, Yuhong Chen, Wanxiu Hai

**Affiliations:** 1 College of Materials Science and Engineering, North Minzu University, Yinchuan 750021, China; matinal757791@163.com (S.Z.); falianqin@163.com (F.Q.); 19111979083@163.com (M.G.); w12449596@163.com (Z.W.); meilingliu@nmu.edu.cn (M.L.); lyhchen@163.com (Y.C.); 2 Key Laboratory of Powder Materials & Advanced Ceramics, North Minzu University, Yinchuan 750021, China

**Keywords:** high-entropy carbides, multiphase ceramics, friction and wear properties

## Abstract

High-entropy carbide (NbTaTiV)C_4_ (HEC4), (MoNbTaTiV)C_5_ (HEC5), and (MoNbTaTiV)C_5_-SiC (HEC5S) multiphase ceramics were prepared by spark plasma sintering (SPS) at 1900 to 2100 °C, using metal carbide and silicon carbide (SiC) as raw materials. Their microstructure, and mechanical and tribological properties were investigated. The results showed that the (MoNbTaTiV)C_5_ synthesized at 1900–2100 °C had a face-centered cubic structure and density higher than 95.6%. The increase in sintering temperature was conducive to the promotion of densification, growth of grains, and diffusion of metal elements. The introduction of SiC helped to promote densification but weakened the strength of the grain boundaries. The average specific wear rates for HEC4 were within an order of magnitude of 10^−5^ mm^3^/N·m, and for HEC5 and HEC5S were within a range of 10^−7^ to 10^−6^ mm^3^/N·m. The wear mechanism of HEC4 was abrasion, while that of HEC5 and HEC5S was mainly oxidation wear.

## 1. Introduction

As a kind of high-entropy ceramic, high-entropy carbide (HEC) shows excellent properties, such as a high melting point, high strength, and high hardness. It is widely used in ultra-high temperature structural components and wear-resistant materials [1,2,3].

HECs are prepared with a variety of methods [3,4], such as solid solution reaction of metal carbides [2,3,4,5,6,7], carbonization reaction of metals [8,9], and carbothermic reduction of oxides [10,11]. In carbonization, metals are prone to be oxidized. In carbothermal reduction, the content of carbon plays a role in adjusting the structure and properties of products [1,4]. In contrast, synthesis via a solid solution reaction seems to be a simple and convenient method. Using self-synthesized carbide powders as raw materials, (TiZrHfVNbTa)C with different vanadium contents were fabricated by pressureless sintering at 2300 °C to 2500 °C [12]. The (TiZrHfVNbTa)C sintered at 2300 °C achieved a density of 97.5 % and a homogeneous microstructure. The bending strength and Vickers hardness were 473 MPa and 24.9 GPa, respectively [12]. (Hf_0.2_Ta_0.2_Zr_0.2_Nb_0.2_Ti_0.2_)C was hot pressed at 1800–1950 °C for 30 min under 30 MPa. A relative density higher than 99% was achieved. The bending strength, compressive strength, fracture toughness, and hardness for the sample sintered at 1850 °C were 494 MPa, 1181 MPa, 2.3 MPa·m^1/2^, and 24 GPa, respectively [13]. (HfTaZrTi)C and (HfTaZrNb)C with a high purity, high density (99%), and chemical homogeneity were fabricated using ball milling and spark plasma sintering (SPS) at 1800 °C and 2300 °C. The nanohardness of (HfTaZrNb)C was 36.1   GPa, which was higher than the hardest monocarbide, HfC (31.5  GPa), and the binary (Hf-Ta)C (32.9   GPa) [3]. It was concluded that the temperature for densification of HECs is relatively high.

In our previous work, a dense (VNbTaMoW)C_5_ was prepared at relatively low temperatures from 1600 °C to 2200 °C [14]. With SPS at 1900 °C for 12 min, the element distribution of (VNbTaMoW)C_5_ was uniform and its fracture toughness was 5.4 MPa·m^1/2^. By adding a secondary phase, the densification and strength of the high-entropy carbide could be promoted [15,16,17]. Lu et al. reported a multiphase ceramic of (TiZrHfNbTa)C-20SiC with increased density, strength, and toughness. The coarsening of grains was inhibited by SiC [15]. Wei et al. reported a refined microstructure and enhanced thermal conductivity of (Ti_0.2_Zr_0.2_Hf_0.2_Nb_0.2_Ta_0.2_)C–graphite ceramics. The incorporated graphite promoted the densification behavior, refined the grains, and improved the mechanical properties of the HEC ceramics [16].

High-entropy carbide shows excellent wear resistance. Coupled with cemented carbide (WC, Co bonded) at room temperature, the average coefficients of friction (CoF) of (MoNbTaVW)C_5_ and (HfNbTaTiZr)C_5_ are as low as 0.25 and 0.36, respectively [14], and their average specific wear rates (WRs) are within orders of magnitude of 10^−6^ mm^3^/N·m. The reported CoF is much lower than that of traditional non-oxides ceramics (such as SiC and Si_3_N_4_) under the same conditions. With the addition of SiC, multiphase ceramics (MoNbTaVW)C_5_-SiC show better fracture toughness (K_1c_, 5.7 MPa·m^1/2^) and anti-wear properties (WRs, 10^−8^ mm³/N·m) [17].

In this paper, a high-entropy carbide of (MoNbTaTiV)C_5_ was prepared by SPS at a relatively low temperature. The effects of sintering temperature and the addition of SiC on the microstructure and properties of (MoNbTaTiV)C_5_ were investigated.

## 2. Experimental Section

### 2.1. Preparation

The particle size and composition of raw carbide powders are shown in Table 1. The carbide powders were weighed according to the composition of HECs as listed in Table 2.

Carbide powders are put into a cemented carbide (WC, Co bonded) mill tank, adding ethanol and cemented carbide grinding balls. The ratio of balls to powder was 5:1. The mixture was ball milled for 8 h. The mixed slurry was dried at 100 °C in an oven. The mixed powder was put into a graphite mold with an inner diameter of 25 mm. Then it was sintered by SPS (SPS-4, Shanghai Chenhua technology co., Shanghai, China) from 1900 °C to 2100 °C under 40 MPa for 12 min. The heating rate was 120 °C/min from 25 °C to 1600 °C, while the pressure was increased linearly from the minimum pressure (10 MPa) to 30 MPa. From 1600 °C to the setting sintering temperature (1900 °C to 2100 °C), the heating rate was 60 °C/min, while the pressure was increased linearly from 30 MPa to 40 MPa. The sample was held at the sintering temperature and 40 MPa for 12 min. Then the sample was cooled to 1000 °C at a rate of 100 °C/min, with the pressure released to 10 MPa. Finally, the sample was cooled naturally. The as-sintered HECs were named HEC4, HEC519, HEC520, HEC5205, HEC521, and HEC5S according to their composition and sintering temperature, as shown in Table 2.

### 2.2. Measurement and Analysis

The phase of the HECs was analyzed by X-ray diffraction (XRD, XRD-6000, Shimazu, Japan), using Cu-Kα radiation and a scanning rate of 2°/min. The microstructure was evaluated using a scanning electron microscope (FESEM, Sigmas, Zeiss, Germany) equipped with an X-ray energy disperse spectrometer (EDS, Oxford, UK). The average grain size was measured using the linear intercept method using FESEM images. More than 150 grains were measured for each sample. The density of the HECs was measured using Archimedes’ principle. The lattice parameters and theoretical density of the HECs were calculated according to XRD patterns, using a method described at length in reference [18]. The hardness was tested with a Vicker’s hardness tester at 98 N for 10 s. Each sample was tested 10 times. The nanohardness and Young’s modulus of the HECs were measured using a nanoindenter (UNHT, CSM Instruments Co., Neuchâtel, Switzerland) at 8 mN. The Poisson ratio was 0.25. Each sample was tested 30 times. The fracture toughness (K_1C_) was determined with the microcrack method according to Anstis [19] (Equation (1)). Each sample was tested 10 times.

(1) $$K_{1C}=0.016{\left(\frac{E}{H}\right)}^{1/2}\frac{P}{c^{3/2}}$$

Friction and wear tests were conducted at room temperature on a unidirectional tribo-tester (HT-1000, Lanzhou Zhongke Kaihua Instrument Co. Ltd., Lanzhou, China). The HECs were machined into disks with a diameter of 25 mm and a thickness of 8 mm. A commercial cemented carbide (WC, Co bond) with a diameter of 5 mm was used as the counterpart material (ball). The load and sliding velocity were 15 N and 0.3 m/s, respectively. The test duration was 1 h. Each sample was tribotested 3 times. The CoF of each test was the average value of CoF in the stable stage. The reported average CoF was the mean value of 3 independently measured values. The WRs was calculated according to Equation (2) by measuring the wear mass loss [14].

W = V/FL(2)

The worn surface of HECs was analyzed with scanning electron microscopy (FESEM, Sigmas, Zeiss, Germany). The composition of the as-polished surface and worn tracks on the HECs were analyzed with X-ray photoelectron spectroscopy (XPS, ThermoFisher 250Xi, Waltham, MA USA).

## 3. Results and Discussion

### 3.1. XRD

**Figure 1:**
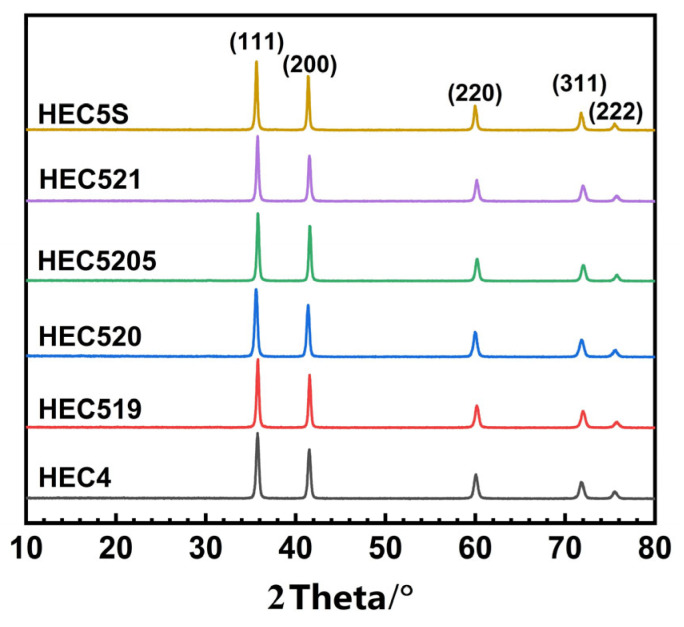
XRD pattern of HECs.

Figure 1 shows the XRD pattern of HECs. In Figure 1 shows the as-sintered (NbTaTiV)C_4_ and (MoNbTaTiV)C_5_ as single phases of the fcc lattice. In a 2θ value range of 10° to 80°, the five peaks were the (111), (200), (220), (311), and (222) diffractions of the *fcc* lattice [2]. With the addition of SiC, the XRD pattern of the as-prepared HEC5s was similar to that of HEC and HEC5. However, the structure of HEC5S is unclear, due to the missing diffraction peaks designated to SiC. It has been reported that the peak intensity of SiC is much lower than that of metal carbides in XRD patterns, which results in the missing diffraction peaks belonging to SiC [15,17].

The relative density of the HECs is shown in Table 3. The lattice parameter of HEC was calculated using the diffraction peaks in the XRD patterns. The theoretical density of the ceramics was calculated from the lattice parameter. The lattice parameter of HEC4 was 4.352 Å, and the corresponding theoretical density was 8.474 g/cm^3^. With the addition of Mo_2_C, the lattice parameter of the as-synthesized HEC5 was in the range of 4.341 to 4.350 Å, and the corresponding calculated theoretical density was in the range of 8.53 to 8.59 g/cm^3^. Compared to HEC4, a slight decrease in lattice parameter and an increase in the calculated theoretical density of HEC5 resulted from a solid solution of Mo in the high-entropy system. The atomic radius of Mo (0.136 nm) was nearly equivalent to V (0.135 nm), which is the smallest among metal atoms forming HEC4 [20].

### 3.2. Microstructure

The lattice parameters and density of the HECs are shown in Table 3. The relative density of HEC519 was 95.6%, and its open porosity was 0.13%. By increasing the temperature, the relative density of HEC5 increased, while the open porosity remained unchanged (0.12%). The relative density of HEC5205 was 96.1%. By the addition of SiC, the relative density of HEC5S increased to 98.8%. By contrast, the relative density of HEC4 sintered at the same temperature was only 95.0%. From the above, densification of HEC was facilitated by increasing the sintering temperature, number of metal components, and the addition of SiC.

**Figure 2:**
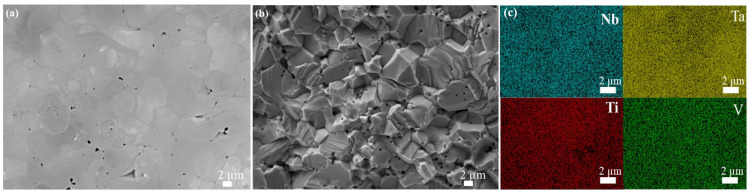
SEM images and EDS mapping of (NbTaTiV)C_4_, (**a**) polished surface, (**b**) fractured surface, and (**c**) elemental mapping of (**a**).

**Figure 3:**
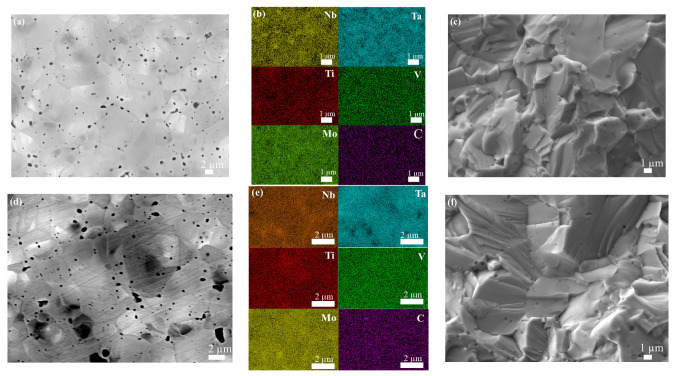

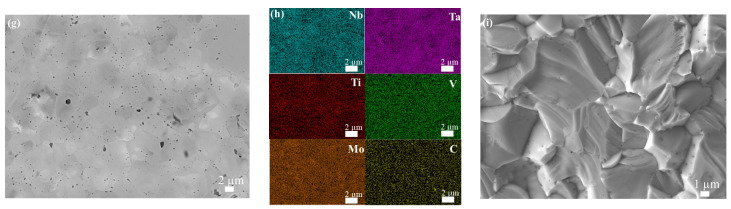
SEM images of (MoNbTaTiV)C_5_: (**a**) HEC519 polished surface, (**b**) corresponding elemental mapping of metal elements in (**a**,**c**) HEC519, fractured surface, (**d**) HEC520 polished surface, (**e**) corresponding elemental mapping of metal elements in (**d**,**f**) HEC520 fractured surface, (**g**) HEC5205 polished surface, (**h**) corresponding elemental mapping of metal elements in (**g**,**i**) HEC5205 fractured surface.

SEM images and the elemental mapping by EDS of the HECs are shown in Figure 2 and Figure 3. In Figure 2a, a small number of pores exist along the grain boundaries and in the grains of HEC4. This could be interpreted that most of the existing pores are closed, as the open porosity of HEC4 was as low as 0.11% (Table 3). The average grain size of HEC4 was 2.4 μm. In Figure 2b, the fracture modes of HEC4 are transgranular and intergranular, and the pores between grains and within grains are spherical. In Figure 2c, the four metal elements and C are distributed uniformly, indicating that all elements were fully diffused without any segregation at 2050 °C.

In Figure 3, there are obvious pores at the grain boundaries and within grains in HEC5. Similarly, most of the pores are closed ones, since the open porosity of HEC5 was in the range of 0.11% to 0.13% (Table 3). In Figure 3c,f,i, there are a small number of nanosized spherical particles, with the particle size and number decreasing with increasing sintering temperature, which might be due to metal carbides that did not participate in the solid solution reaction [21]. The average grain sizes of HEC519, HEC520, and HEC521 were 1.9 μm, 2.1 μm, and 3.8 μm, respectively. The grain size of HEC21 was 7.6 μm. The fracture mode of the HECs (Figure 3b,d,f) was mainly transgranular. In HEC519 and HEC520, the Ti and V were distributed evenly, while Ta, Mo, and Nb showed slight segregation [22]. In HEC205, all elements were distributed evenly.

**Figure 4:**
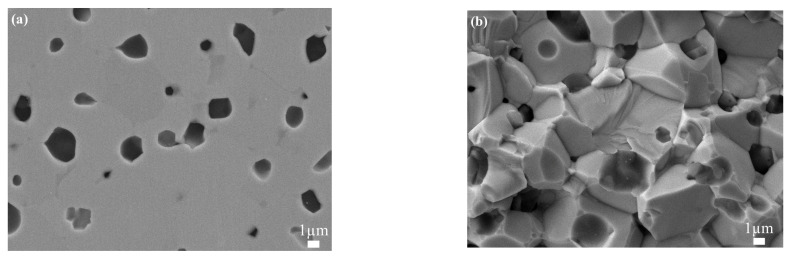
SEM images of (MoNbTaTiV)C_5_-10% SiC (**a**) polished surface and (**b**) fractured surface.

SEM images of the polishing and sectional surface of HEC5S are shown in Figure 4. As can be seen from Figure 4a, the HEC phase is continuous and the SiC particles are distributed evenly along the grain boundaries. Thus, by adding SiC, multiphase ceramics of HEC and SiC were formed. The average grain size of the HEC phase in HEC5S was 3.8 μm (Table 3), which is comparable to that of HEC5205. In Figure 4b, the fracture mode of multiphase ceramics is mainly intergranular, which is different from the transgranular fracture mode of single-phase HECs in Figure 3. It can be concluded that the introduction of SiC weakened the grain boundary strength of the multiphase ceramics. Compared with Figure 3 and Figure 4, the addition of SiC weakened the grain boundary strength of HECs at the same sintering temperature.

From the above, the density and grain size of HECs were elevated by increasing the sintering temperature, though closed pores remained inside the HECs. By increasing the number of the metal components and with the addition of SiC, the densification of HECs was promoted.

### 3.3. Mechanical Properties

The mechanical properties of the HECs are shown in Table 4. In Table 4, the Vickers’ hardness of HEC5 increased slightly with the sintering temperature. HEC5205 had the highest hardness of 21.8 GPa. With the increase in temperature, the nanohardness of HEC5 increased slightly and the Young’s modulus increased gradually, while the fracture toughness decreased gradually (related to the gradual growth of the grains) [23]. Compared with HEC4, the hardness, nanohardness, and Young’s modulus of HEC5205 increased, while their fracture toughness was comparable. With the introduction of SiC, the Vickers hardness of HEC5S was lower and the fracture toughness higher than HEC5205. This variation was probably due to the higher relative density of HEC5S (Table 3).

In the literature [24,25,26], *H/E* and *H*^3^*/E*^2^ were adopted to explain fracture toughness. According to Chen [26], a lower *H*/*E* or *H*^3^*/E*^2^ indicates a lower brittleness and higher damage tolerance. In Table 4, the toughness of (MoNbTaTiV)C_5_ was in the range of 3.5 MPa·m^1/2^ to 4.8 MPa·m^1/2^, with the H/E in the range of 0.44 to 0.50, and *H*^3^*/E*^2^ in the range of 0.039 GPa to 0.050 GPa. The *H/E* here was comparable to that of (V_0.2_Nb_0.2_Ta_0.2_Mo_0.2_W_0.2_)C reported by Harrington et al. (0.046) [27] and higher than that of Li et al. (0.036) [28]. The *H/E* or *H*^3^*/E*^2^ of (MoNbTaTiV)C_5_ are positively correlated with their fracture toughness. This might be due to the fracture mode of high-entropy ceramics being purely brittle fracture, without plastic deformation. In addition, the positive correlation here is different from the negative correlation reported by Li [28]. According to Li, (TiVNbMoW)C_4.375_ shows a low *H/E* value, indicating that the high-entropy (TiVNbMoW)C_4.375_ possesses a lower brittleness and higher damage resistance compared to other high-entropy transition metal carbides. It can be seen that the conclusions of this paper and that of Li [28] are inverse. Obviously, more evidence is needed.

### 3.4. Tribological Properties

The average CoF and average WRs of the HECs are listed in Table 5. The average CoF and average WRs of HEC4 were 0.41 and 1.5 × 10^−5^ mm^3^/N·m, respectively. The CoF of HEC519 to HEC521 was in the range 0.44–0.48. The WRs of HEC5 was 1 to 2 orders of magnitude lower than that of HEC4. The WRs of HEC519 to HEC521 first increased and then decreased with increasing sintering temperature. The WRs of HEC519 and HEC521 were as low as 10^−7^ mm^3^/N·m. The average CoF of HEC5S was 0.54, slightly higher than that of HEC4 and HEC5. The average WRs of HEC5S was 2.14 × 10^−6^ mm^3^/N·m. It can be seen that HEC5 and its multiphase ceramics showed a lower WRs than the quaternary HECs and represent wear-resistant materials.

**Figure 5:**
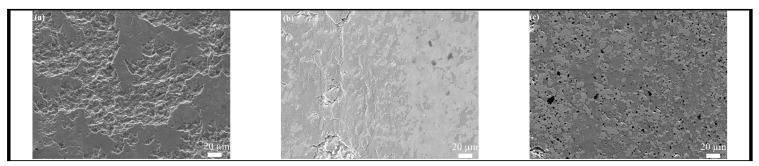
SEM images of worn surface of (**a**) HEC4, (**b**) HEC5205, and (**c**) HEC5S.

SEM images of the worn surface of the HECs are shown in Figure 5. As can be seen from Figure 5a, the worn surface of HEC4 was rough and there are obvious pits formed by the fracturing and pulling-out of grains. Meanwhile, there are compacted tribo-layers distributed discontinuously. Comparatively, the tribo-layers on the worn surfaces of HEC5205 and HEC5S are distributed continuously (Figure 5b,c). The difference is that the tribo-layer of HEC5205 is dense (Figure 5b), while that of HEC5S is loose and the size of debris is in the nanoscale (Figure 5c).

**Figure 6:**
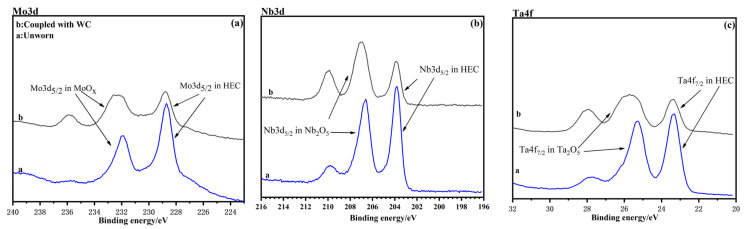

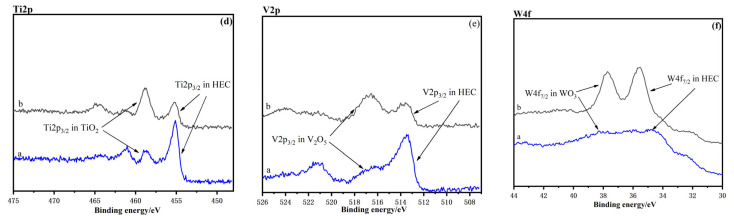
XPS spectra of elements on the worn surface of HEC5205: (**a**) Mo 3d, (**b**) Nb3d, (**c**) Ta4f, (**d**) Ti2p (**e**) V2p, and (**f**) W4f. The letter a represents the as polished surface (unworn surface), while b represents worn tracks as coupled with WC.

The binding energy of elements on the worn surface of HEC5205 is shown in Figure 6. In Figure 6, the peaks at 228.8 eV and 232.6 eV are designated as Mo3d_5/2_ in HEC and MoO_3_, respectively (Figure 6a) [29,30]. The peaks at 203.9 eV and 207.5 eV are assigned to Nb3d_5/2_ in HEC and Nb_2_O_5_, respectively (Figure 6b) [29,31]. The peaks at 23.6 eV and 25.5 eV are assigned to Ta4f_7/2_ in HEC and Ta_2_O_5_, respectively (Figure 6c) [29,32]. The peaks at 455.3 eV and 458.8 eV are assigned to Ti2p_3/2_ in HEC and TiO_2_, respectively (Figure 6d) [29,33]. The peaks at 513.7 eV and 517.2 eV are assigned to V2p_3/2_ in HEC and V_2_O_5_, respectively (Figure 6e) [29,34]. The peaks at 32.5 eV and 35.7 eV are assigned to W4f_7/2_ in HEC and WO_3_, respectively (Figure 6f) [29,35]. It can be seen that compared with the carbides on the unworn surface, the metal elements on the worn surface of HEC5 are mainly metal oxides.

As can be seen from Figure 5 and Figure 6, and Table 4, the wear mechanism of HEC4 was abrasive wear and the tribo-layer played a role in reducing wear. This was also the reason why the CoF and WRs of HEC were smaller under dry conditions than that of the binary metal carbides [28]. The wear mechanism of HEC5 and HEC5S was mainly oxidation wear. Continuous tribo-oxide layers play a role in reducing friction and wear.

## 4. Conclusions

- Dense (MoNbTaTiV)C_5_ with fcc structure was prepared using SPS at 1900 to 2100 °C using metal carbide as raw material. The densification of the HECs was promoted by increasing the number of metal components, increasing the sintering temperature, and the addition of SiC.
- Elevating the temperature, the growth of grains was promoted, resulting in full diffusion of metal elements to achieve a uniform distribution. The introduction of SiC in HECs weakened the strength of the grain boundaries.
- With the increase of sintering temperature, the hardness of HEC5 increased slightly and the fracture toughness decreased gradually.
- The WRs was 10^−5^ mm^3^/N·m for HEC4 and was in the range of (10^−7^–10^−6^) mm^3^/N·m for HEC5 and HEC5S. The wear mechanism of HEC4 was abrasive wear, while that of HEC5 and HEC5S was mainly oxidation wear.

## Figures and Tables

**Table 1:** Characteristics of the starting carbide powders and manufacturer information.

Carbides	Average Particle Size/μm	Purity and Main Impurities/wt%	Manufacturer
Mo_2_C	4.5	Purity >99.6%; 0.008free C, 0.003 Fe, 0.002 S, 0.29 O	Shanghai Yunfu Nano Material Co., LTD, Shanghai, China
NbC	1.21	Purity >99.7%; 11.07 total C, 0.08 free C, 0.02 Ta, 0.19 O	Ningxia Orient Tantalum Industry Co., LTD, Shanghai, China
TaC	1.04	Purity >99.5%; 6.25 total C, 0.05 free C; 0.01 Nb, 0.02 Fe, 0.21 O	Ningxia Orient Tantalum Industry Co., LTD, Shanghai, China
TiC	3.5	Purity >99.5%; 0.0087 free C; 0.0037 Fe, 0.0011 Mg, 0.0023 S, 0.29 O	Shanghai Yunfu Nano Material Co., LTD, Shanghai, China
VC	4.5	Purity >99.5%; 0.008 free C, 0.003 Fe, 0.002 S, 0.3 O	Shanghai Yunfu Nano Material Co., LTD, Shanghai, China
α-SiC	0.6	Purity >98.8%; 0. 03 Al, 0.05 Fe, 0.01 Ca, 1.1 O	S H.C. Starck Surface Technology and Ceramic Powders GmbH, Goslar, Germany

**Table 2:** Composition of HECs and sintering temperature.

No.	Composition	Sintering Temperature /°C
HEC4	(Nb_0.23_Ta_0.24_Ti_0.26_V_0.27_)C	2050
HEC519	(Mo_0.24_Nb_0.20_Ta_0.20_Ti_0.23_V_0.23_)C	1900
HEC520	(Mo_0.24_Nb_0.20_Ta_0.20_Ti_0.23_V_0.23_)C	2000
HEC5205	(Mo_0.24_Nb_0.20_Ta_0.20_Ti_0.23_V_0.23_)C	2050
HEC521	(Mo_0.24_Nb_0.20_Ta_0.20_Ti_0.23_V_0.23_)C	2100
HEC5S	(Mo_0.24_Nb_0.20_Ta_0.20_Ti_0.23_V_0.23_)C-10vol.%SiC	2050

**Table 3:** Lattice parameter, density, and grain size of HECs.

No.	Lattice Parameter /Å	Theoretical Density /g/cm^3^	Measured Density /g/cm^3^	Relative Density /%	Open Porosity /%	Grain Size /μm
HEC4	4.352	8.474	8.050	95.0 ± 0.4	0.11	2.5 ± 1.4
HEC519	4.345	8.562	8.193	95.6 ± 0.8	0.13	1.9 ± 0.9
HEC520	4.350	8.534	8.250	96.0 ± 0.1	0.12	2.1 ± 1.1
HEC5205	4.341	8.585	8.253	96.1 ± 0.5	0.12	3.8 ± 1.8
HEC521	4.346	8.559	8.301	97.0 ± 0.1	0.11	7.6 ± 2.7
HEC5S	4.341	8.024 ^a^	7.928	98.8 ± 0.5	0.16	3.8 ± 1.7

^a^ Theoretical density = ρ(HEC5205) × 90% + ρ(SiC) × 10%.

**Table 4:** Mechanical properties of HECs.

No.	Vicker’s Hardness /GPa	Nanohardness /GPa	Young’s Modulus /GPa	Fracture Toughness /MPa·m^1/2^	H/E	H^3^/E^2^ /GPa
HEC4	20.7 ± 0.91	27.9 ± 0.6	450 ± 70	3.7 ± 0.3	0.046	0.044
HEC519	20.2 ± 0.3	27.9 ± 0.6	405 ± 44	4.8 ± 0.4	0.050	0.050
HEC520	21.0 ± 0.5	27.9 ± 1.2	487 ± 36	3.8 ± 0.3	0.043	0.039
HEC5205	21.8 ± 0.6	28.6 ± 0.7	501 ± 50	3.5 ± 0.2	0.044	0.041
HEC5S	20.5 ± 0.7		492 ± 45	3.8 ± 0.3	0.042	0.036

**Table 5:** Average CoF and average WRs of HECs.

No.	Average CoF	Average WRs /× 10^−6^ mm^3^/N·m
HEC4	0.41 ± 0.08	14.9 ± 5.0
HEC519	0.48 ± 0.05	0.65 ± 0.40
HEC520	0.44 ± 0.08	2.31 ± 0.80
HEC5205	0.48 ± 0.02	1.61 ± 1.20
HEC521	0.47 ± 0.05	0.40 ± 0.05
HEC5S	0.54 ± 0.01	2.23 ± 1.50

## Data Availability

Data sharing is not applicable to this article.
